# Supplementary material for: Nonlinear feedback modulation contributes to the optimization of flexible decision-making
Source: eLife. 2025 Sep 30;13:RP96402. doi: 10.7554/eLife.96402 (PMC12483514; doi:10.7554/eLife.96402)
Supplement: Supplementary file 1. [file elife-96402-supp1.docx]

| Hyperparameter | Value | Hyperparameter | Value |
| --- | --- | --- | --- |
| Training batch size | 1024 | Decision threshold | 0.8 |
| Testing batch size | 2048 | Training stimuli coherence levels | [0.6, 0.9] |
| Learning rate | 0.02 | Testing stimuli coherence levels | [0, 0.35, 0.55, 0.75] |
| Number of input/hidden/output units | 17($N_{in}$), 200($N_{rec}$), 2($N_{out}$) | Fixation/Target/Stimulus/Decision Time | 500ms($T_{fix}$),400ms($T_{targ}$), 500ms($T_{stim}$), 100ms($T_{dec}$) |
| Time step | 20 | Spike cost | 0.004 |
| Membrane time constant | 100 | Weight cost | 1 |
| STSP neurotransmitter time constant | 200ms / 1000ms  (facilitating / depressing) | Input noise distribution std | 0.07 ($\sigma_{in}$) |
| STSP neurotransmitter utilization | 2000ms / 200ms  (facilitating / depressing) | Noise distribution std of hidden unit | 0.08 ($\sigma_{rec}$) |
| STSP neurotransmitter increment | - 1. / 0.3   (facilitating / depressing) |  |  |

**Table S1.** Important model hyperparameters
